# Mortality impact of an increased blood glucose cut-off level for hypoglycaemia treatment in severely sick children in Malawi (SugarFACT trial): study protocol for a randomised controlled trial

**DOI:** 10.1186/s13063-017-2411-8

**Published:** 2018-01-11

**Authors:** Tim Baker, Queen Dube, Josephine Langton, Helena Hildenwall

**Affiliations:** 1 Global Health – Health System and Policy Research Group, Department of Public Health Sciences, Karolinska Institutet, 171 77 Stockholm, Sweden; 2 Department of Anaesthesia & Intensive Care, Queen Elizabeth Central Hospital, Blantyre, Malawi; 3 Perioperative Medicine and Intensive Care, Karolinska Univeristy Hospital, Stockholm, Sweden; 4 Department of Paediatrics, College of Medicine, University of Malawi, Blantyre, Malawi; 5 Astrid Lindgren Children’s Hospital, Karolinska University Hospital, Stockholm, Sweden

**Keywords:** Hypoglycaemia, Critical care, Paediatrics, Emergency medicine

## Abstract

**Background:**

Mortality in children remains high in sub-Saharan African hospitals. While antimalarial drugs, antibiotics and other definitive treatments are well understood, the role of emergency care with supportive therapies, such as maintaining normal glucose and electrolyte balances, has been given limited attention. Hypoglycaemia is common in children admitted to hospital in low-income settings. The current definition of hypoglycaemia is a blood glucose level < 2.5 mmol/L in a well-nourished child. Outcomes for these children are poor, with a mortality rate of up to 42%. An increased mortality has also been reported among acutely ill children with low-glycaemia, defined as a blood glucose level of 2.5–5.0 mmol/L. The reason for increased mortality rates is not fully understood. This proposal is for a randomised controlled trial to determine the impact on mortality of a raised treatment cut-off level for paediatric hypoglycaemia.

**Methods:**

A total of 1266 severely ill children (age range = 1 month – 5 years) admitted to Queen Elizabeth Central Hospital in Blantyre, Malawi with blood glucose in the range of 2.5–5.0 mmol/L will be randomised into intervention or control groups. The intervention group will be treated with an intravenous bolus of 10% dextrose 5 mL/kg followed by a dextrose infusion in addition to standard care while the control group will receive standard care only. Children will be followed until discharge from hospital or death.

**Discussion:**

The first patient was enrolled in December 2016 and the expected trial deadline is January 2019. This study is the first to evaluate the benefits of increased dextrose administration in children presenting to hospital with low-glycaemia. The findings will inform national and international policies and guidelines for the management of children with blood sugar abnormalities.

**Trial registration:**

ClinicalTrials.gov, NCT02989675. Registered on 5 December 2016.

**Electronic supplementary material:**

The online version of this article (10.1186/s13063-017-2411-8) contains supplementary material, which is available to authorized users.

## Background

More than six million children aged < 5 years die each year in low-income countries, the majority due to treatable infectious diseases with pneumonia, diarrhoea and malaria as the main killers [1]. While the management of children in African hospitals and health centres routinely involves the administration of antimalarial drugs, antibiotics and other definitive treatments, the role of emergency care with supportive treatments, such as oxygen, fluids and glucose, has received less attention [2, 3]. Hypoglycaemia is known to be associated with poor clinical outcome and long-term negative effects on neurodevelopment [4]. Most low-income settings lack the capacity for rapid laboratory confirmation of hypoglycaemia and the presenting symptoms are often unspecific, such as drowsiness, agitation, hypothermia and seizures. The World Health Organization (WHO) currently defines paediatric hypoglycaemia as a blood glucose level < 2.5 mmol/L (or < 3 mmol/L in a severely malnourished child) [5]. A blood glucose level above these limits is considered adequate for sufficient energy supply for vital functions and particularly to the brain, which is dependent on glucose metabolism.

Previous studies have shown that 1.8–7.3% of children admitted to hospital in sub-Saharan Africa are hypoglycaemic [6–9]. In a recent study from north-eastern Tanzania, hypoglycaemic children had an increased risk of death with a mortality rate of as much as 42% [6]. That study and others in similar settings have also showed an increased mortality in children with low-glycaemia, defined as a blood glucose level of 2.5–5.0 mmol/L, leading to a questioning of the current cut-off for hypoglycaemia [6, 9, 10]. In children with an emergency sign, as defined in the WHO Pocket Book of Hospital Care for Children [5], and low-glycaemia, the mortality was 15% compared to 6% mortality for those with an emergency sign and blood glucose > 5.0 mmol/L (personal communication Nadjm B 2015).

Plasma glucose concentration is maintained by the interplay of the glucose-lowering action of insulin and the glucose-raising actions of the four counter-regulatory hormones cortisol, catecholamines, glucagon and growth hormone. The counter-regulatory response has been shown at plasma levels ≤ 3.8 mmol/L [11]. In acute illness, the normal physiological response is the release of stress hormones such as cortisol, which activates the release of glucose into the blood, consequently increasing the blood glucose levels and causing hyperglycaemia.

Paediatric neuro-endocrine responses in critical illness have not been studied in detail, although several differences in the host response in children compared to adults have been suggested [12]. The ‘natural hyperglycaemia’ occurring in critical illness due to the release of stress hormones could potentially be beneficial: the higher glucose concentration compensating for a decreased or dysfunctional circulation. The child with low-glycaemia may, therefore, be a result of late care-seeking or more severe illness leading to an abnormal stress response [13] and a ‘pre-hypoglycaemic stage’. Alternatively, the lack of an increased blood glucose level may be a result of failure to use alternative sources of energy such as ketone bodies [14], infection with specific pathogens, prolonged fasting during the illness period and/or a sub-optimal nutritional status before the illness. These children may benefit from increased glucose administration.

The current cut-off level for treating hypoglycaemia (<2.5 mmol/L for well-nourished children or < 3.0 mmol/L for severely malnourished children) is based on pathophysiological assumptions that lack robust evidence. This study will report the effect of therapeutically targeting low-glycaemia, in other words the study tests raising the cut-off for treating low blood glucose from < 2.5 mmol/L to < 5.0 mmol/L.

## Methods

### Specific objectives

I. To determine the impact on in-hospital mortality of administering dextrose to severely ill children aged 1 month to 5 years with low-glycaemia at arrival to the emergency department
II. To determine the impact on 24-h mortality of administering dextrose to severely ill children aged 1 month to 5 years with low-glycaemia at arrival to the emergency department

### Trial design

This is a single-centre, non-blinded, two-arm randomised controlled trial that will compare the impact of dextrose treatment in children with WHO emergency signs admitted to hospital with low-glycaemia. The present paper is written according to the Standard Protocol Items: Recommendations for Interventional Trials (SPIRIT) 2013 Statement for reporting a clinical trial protocol [15–17]. The SPIRIT Checklist is provided as Additional file 1.

### Trial setting

Queen Elizabeth Central Hospital is a large university hospital in Blantyre, Malawi. The hospital serves a predominately rural population and manages 23,000 paediatric admissions annually (personal communication Molyneux E 2015). The Emergency Triage, Assessment and Treatment (ETAT) protocol [18] was developed in Queen Elizabeth Hospital and is used as the standard for management of sick children. Children will be recruited from the Paediatric Emergency Department at Queen Elizabeth Central Hospital.

### Participants

All children arriving to the emergency department for admission to Queen Elizabeth Central Hospital in Blantyre, Malawi will be screened for the presence of the following inclusion and exclusion criteria.

#### Inclusion criteria

- Age range of one month to 5 years
- Parent/carer willing and able to give consent
- Presence of one or several emergency signs [5]^1^
  - ○ Obstructed or absent breathing
  - ○ Central cyanosis
  - ○ Severe respiratory distress
  - ○ Shock/impaired perfusion
  - ○ Coma/reduced consciousness
  - ○ Convulsions
  - ○ Severe dehydration
  - ○ Clinical concern that the child is in an emergency state
- Blood glucose 2.5–5.0 mmol/L at arrival to the emergency department (3.0–5.0 mmol/L for severely malnourished children)

#### Exclusion criteria

- Children with a known diagnosis of diabetes
- Refusal to participate by the child

### Blood glucose testing

Capillary blood glucose levels will be measured using the HemoCue Glucose 201 point of care device, which has shown reliable performance [19]. Quality control will be done by regular controls with standardised glucose fluids.

### Randomisation and group allocation

Randomisation will be to the two arms of the trial: the intervention arm and the control arm. Children with severe malnutrition will be randomised separately in another stratum to ensure the same number of children in each arm of each stratum. An independent statistician who is not part of the research team has produced a computer-generated randomisation list using a two-stage process to ensure allocation concealment. The computer first randomly selects whether the block will contain six or eight patients and then randomly chooses one of the possible different allocation blocks for the selected size.

The investigators assign the subject identification numbers sequentially and group allocation is determined when the research staff opens an opaque envelope prepared in advance by the independent trial statistician. Envelopes are opened in the emergency department as soon as a child has been identified as eligible for the trial. The study identification number will be retained throughout the study and will appear on all case report form pages and source documents.

### Sample size calculations

The required sample size using the following assumptions:

- a 1:1 intervention: control ratio
- A power of 0.80
- Significance level 0.05
- Mortality in control group: 15.4%
- Mortality in intervention group: 10.0%

is calculated to be 1266 children aged < 5 years, 633 in each arm. Assuming that 35% of children with an emergency sign present with low-glycaemia (unpublished data from Queen Elizabeth Central Hospital), and around 10% will refuse to participate, the total number to be screened is 4000.

### The intervention

Children in the intervention group will immediately receive a bolus of intravenous 5 mL/kg 10% dextrose, prepared using one part 50% dextrose and four parts 0.9% sodium chloride, (Ringer’s Lactate will be used instead of sodium chloride in severely malnourished children, in accordance with current local procedures). If intravenous cannulation is not possible after 15 min of attempting cannulation, then an intraosseous needle will be sited and used until intravenous cannulation is possible. Dextrose administration will continue as a maintenance infusion of intravenous 10% dextrose for 24 h, prepared using one part 50% dextrose and four parts 0.9% sodium chloride (Ringer’s Lactate for malnourished children) at standard maintenance rates (over 24 h: 100 mL/kg for the first 10 kg body weight, then 50 mL/kg for the next 10 kg, thereafter 25 mL/kg for each subsequent kg). If the clinical condition allows, the child will be given oral nutrition in accordance with current local procedures. Capillary blood glucose monitoring will be repeated at 30-min intervals, in line with ETAT recommendations for hypoglycaemia, with repeated equivalent doses given until levels reach ≥ 5.0 mmol/L. Severely malnourished children will be treated according to WHO guidelines with nasogastric rehydration as the first option, in accordance with local procedures.

**Fig. 1:**
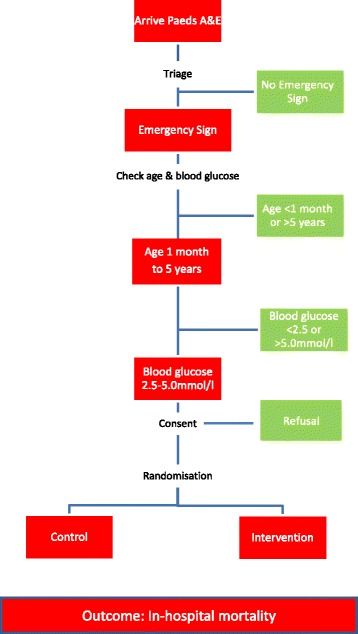
Study *flowchart*

All children in the control group will be kept in the emergency department for a minimum of 60 min and have their vital signs checked at discharge from the emergency room to the ward. If their clinical condition deteriorates they will be re-examined by clinical staff. Re-checking of blood glucose may be done if clinically warranted and, if found, hypoglycaemic dextrose infusion in a control child will be documented. The total amount of dextrose given during the admission will be documented for all enrolled patients to allow for comparison of received dextrose between control and intervention groups. Usual care—the care that is currently provided in the hospital—will be provided. This includes care according to local departmental guidelines that are based on the WHO’s ETAT guidelines [18] and definitive treatment of infective causes with antibiotics and/or antimalarial drugs. In contrast to the intervention group, children with low-glycaemia will not receive any bolus dextrose or routine re-checking of their blood glucose level. Figure 1 provides a flowchart of the study.

### Primary and secondary outcome measures

**Fig. 2:**
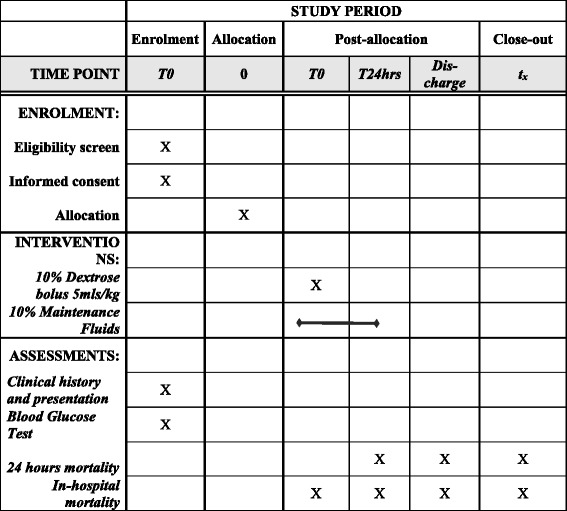
SugarFACT schedule of enrolment, interventions and assessments

The primary outcome is in-hospital mortality. Secondary outcome is 24-h mortality. Follow-up will continue until discharge from hospital or death. The expected trial target is superiority of dextrose treatment in the intervention group compared to the control group. Figure 2 provides a schedule of enrolment, interventions and assessments in the study, according to SPIRIT guidelines.

### Data collection and management

Electronic clinical record forms (CRFs) have been developed from the paper drafts with the assistance of Malawi College of Medicine Research Support Centre’s Data Management team using Open Data Kit (https://opendatakit.org). Data are collected on Android tablets and uploaded to a database on the day of collection. The data management team provides the investigators with weekly data reports including numbers enrolled and any problems in data collection including serious adverse events (SAEs) and protocol violations. Details about the data management plan are in the Data Management Standard Operating Procedures, which can be requested from the study team. Data access will be granted to investigators and the assigned study statisticians with whom a Mutual Confidential Disclosure Agreement has been signed (available upon request from corresponding author). Data will be exported for analysis to a statistics software package such as STATA v 12 for analysis. Principal analysis will be on an intention-to-treat principle and all statistical analysis will be done by a statistician who is independent from the study research team.

### Analysis plan

Collected data will undergo the following hypotheses testing and analysis:

I. H_0_: Children in the intervention group have the same in-hospital mortality as children in the control group.

Analysed by intention-to-treat using logistic regression at a significance level of 0.05. Sub-group analyses will be performed on the a priori-defined groups

II. H_0_: Children in the intervention group have the same 24-h mortality as children in the control group.

Analysed by intention-to-treat using logistic regression at a significance level of 0.05. Sub-group analyses will be performed on priori-defined groups. Details about the analysis are in the Statistical Analysis Plan, which can be requested from the study team.

### A-priori defined sub-groups

#### Sub-group for stratified randomisation

- Severe malnutrition: mid-upper arm circumference < 115 mm (< 110 mm if aged < 6 months) or clinician’s diagnosis (oedema of both feet, severe wasting, hair changes, dermatitis)

#### Sub-groups for sub-group analyses

- Body temperature ≥ 38.5 °C at admission
- Age < 12 months
- Age < 24 months
- Blood glucose < 3 mmol/L
- Blood glucose < 4 mmol/L
- Confirmed malaria by blood slide
- Confirmed bacterial infection by positive blood culture
- Inability to feed before admission
- Guardian’s report of poor nutritional intake before admission
- Trauma or surgery as reason for admission
- Severe acute malnutrition

Primary analysis will be by intention-to-treat. Per-protocol analyses will also be conducted and the number of children who do not follow protocol will be reported. Protocol violations include, in the intervention group: failure to give an initial dextrose bolus; failure to repeat the blood glucose test after 30 min; failure to give a repeat dextrose bolus; failure to prescribe or to start the dextrose infusion or oral nutrition; incorrect dose of dextrose. And in the control group: admission to the ward < 60 min after enrolment; additional blood glucose testing or dextrose administration in the emergency department. Protocol violations are included in the weekly data report from the data manager.

### Blinding

Blinding of group allocation is not possible in the emergency department for the study participants, parents/carers or research staff as ethics preclude the administration of placebo intravenous fluid boluses. Data will be blinded to the data analysist by labelling the groups with non-identifying terms until data collection has been completed. Breakage of blinding will be possible for the Data Safety and Monitoring Board (DSMB) in the interim. A list of the randomisation code will be kept by the independent statistician in Malawi who can communicate with the data manager and the DSMB when needed. A sealed envelope containing the randomisation code will be kept in the investigator site file.

### Trial monitoring and safety

Trial monitoring will be done by an independent clinical trial monitoring team at pre-decided scheduled visits: before study-start; after the enrolment of 20 patients; at 50% enrolment; and at the end of the study. A three-member sponsor-independent DSMB who are not involved in the design or conduct of the study has been appointed to deal with any safety issues that arise while the trial is in progress and to scrutinise the interim analysis. The DSMB consists of one epidemiologist, one professor in paediatrics and one professor in biostatistics. SAEs are identified by the study team and reported to an independent Clinical Monitor within 48 h. Following the Clinical Monitor’s assessment, the SAEs are sent to the DSMB and to the College of Medicine Research and Ethics Committee. Interim analyses will be conducted by the trial statistician and reviewed by the DSMB. The intention is for an analysis conducted at 50% of information time (percentage of participants with data for the primary endpoint) though the DSMB committee may suggest other or additional times for interim analyses. The interim analysis will be done for trial safety including review of eligibility and treatment, summary of response, survival and any adverse events (AEs). Stopping rules are detailed in the Statistical Analysis Plan, which can be requested from the study team. Any important protocol modifications will be communicated to the IRB, the DSMB and the trial registry.

## Discussion

The risk of AEs due to dextrose administration is low and is mainly related to complications of cannulation such as infections or subcutaneous fluid administration. The skin will be disinfected before pricking and the cannula removed in case of signs of subcutaneous fluid administration. Dextrose is a sugar that is rapidly metabolised to carbon dioxide and water in the body, providing energy. The administration of 10% dextrose may cause irritation to blood vessels with an associated discomfort that is self-limiting. Any SAEs occurring in participants, whether likely to be due to the trial or not, will be systematically recorded and reported.

The use of intraosseous needles will be applied in situations of difficult intravenous cannulation. Considering that all study children present with one or many WHO Emergency sign(s), it is considered clinically warranted to ensure a soon access to the systemic venous system, but will only be applied after repeated attempts of intravenous cannulation. In cases of intraosseous cannulation, standard procedures will be applied to avoid complications such as proper cleaning and removal of the needle not later than 24 h after insertion.

Knowledge about the impact of a raised blood glucose cut-off level for dextrose administration in acutely ill children will inform local, national and global management guidelines, potentially improving survival rates.

Study findings will be communicated with the hospital management, the College of Medicine and the Malawian Ministry of Health as soon as available. Results will be further disseminated through scientific publications, presentations at international child health scientific conferences and to the WHO. Trial presentations will adhere to the CONSORT statement [20]. If the findings have public interest, they will be disseminated through newspapers and radio broadcasting

### Trial status

Enrolling since 5 December 2016. At the time of manuscript submission on 5 April 2017, 60 patients (4.8% of the final sample size) had been randomised.

## Additional files

Additional file 1: SPIRIT check list. (DOCX 62 kb)

Additional file 2: Study definitions of emergency signs. (DOCX 18 kb)

Additional file 3: Study consent form (in English). (DOCX 31 kb)

## Abbreviations

COMREC: College of Medicine Research and Ethical Committee
CRF: Clinical record forms
DSMB: Data Safety and Monitoring Board
ETAT: Emergency Triage Assessment and Treatment
IRB: Institutional review board
ODK: Open data kit
SAE: Serious adverse events
SPIRIT: Standard Protocol Items, Recommendations for Interventional Trials
WHO: World Health Organization

## Footnotes

1 Detailed study definitions of emergency signs are available in Additional file 2.
