# Supplementary material for: Mortality impact of an increased blood glucose cut-off level for hypoglycaemia treatment in severely sick children in Malawi (SugarFACT trial): study protocol for a randomised controlled trial
Source: Trials. 2018 Jan 11;19:33. doi: 10.1186/s13063-017-2411-8 (PMC5765642; doi:10.1186/s13063-017-2411-8)
Supplement: Supplementary file 2 — Study definitions of emergency signs. (DOCX 18 kb) [file 13063_2017_2411_MOESM2_ESM.docx]

**Additional file 2: Study Definitions of Emergency Signs**

- Obstructed or absent breathing = one of:
  - Stridor
  - Snoring or gurgling sounds
  - No breathing
  - Slow breathing (Rate per min: 1/12-12/12 <20; 12/12-12yrs <15)
- Central cyanosis / hypoxemia = one of:
  - Blue mucous membranes
  - Oxygen saturation <90%
- Severe respiratory distress = one of:
  - Very fast breathing (Rate per min: 1/12-12/12 >= 60; 12/12-5yrs >= 50; 5-12 yrs >=40)
  - Chest in-drawing
  - Head nodding
  - Nasal Flaring
  - Tracheal Tug
- Shock / impaired circulation = one of:
  - Weak and fast pulse (Rate per min: 1/12-12/12 >= 180; 12/12-12yrs >= 160)
  - Cap refill > 3 seconds
  - Cold hands
  - Hypotension (Systolic BP (mmHg): 1/12-12/12 < 70; 12/12-12yrs < (70 + age/2))
- Coma / reduced conscious level
  - Blantyre Coma Scale <5
- Convulsions
- Severe dehydration = diarrhoea plus two of
  - Reduced skin turgor (>2 seconds)
  - Sunken eyes
  - Lethargy
  - Unable to drink or drinks poorly
- Clinical concern
  - Health worker concerned that the child is an emergency
