# Supplementary material for: Mortality impact of an increased blood glucose cut-off level for hypoglycaemia treatment in severely sick children in Malawi (SugarFACT trial): study protocol for a randomised controlled trial
Source: Trials. 2018 Jan 11;19:33. doi: 10.1186/s13063-017-2411-8 (PMC5765642; doi:10.1186/s13063-017-2411-8)
Supplement: Supplementary file 3 — Study consent form (in English). (DOCX 31 kb) [file 13063_2017_2411_MOESM3_ESM.docx]

**Additional file 3**

**CONSENT INFORMATION**

**Sugar Requirements in Febrile African Children Trial**

**(SugaRFACT)**

**Patient Information Sheet**

**Who are the researchers and what is the research about?**

This is a study being conducted by Dr Queen Dube from the Paediatric Department of Queen Elisabeth Central Hospital, Blantyre Malawi and Dr Helena Hildenwall and Dr Tim Baker from Karolinska Institutet in Sweden.

Many children who are admitted with a febrile illness have a low blood sugar when they arrive to the hospital. Some of these children are currently treated with additional sugar but it is not known at what precise levels treatment with additional sugar is beneficial for the sick child. This study is aiming to find out more about when treatment with additional sugar is needed and the reasons for why children with fever present with low blood sugar. To do this, some children with a blood sugar level within a certain interval will be asked to participate in this study. Some of them will receive additional sugar and some of them will not and we will then compare if any of the groups were doing better than the other.

**What does the study involve for my child?**

Your child will be examined by qualified research and ordinary hospital staff who will treat your child as soon as possible. In addition to the tests performed within the hospital care, we will take a small amount of blood from a finger prick to measure the blood glucose level. Depending on the value of blood sugar in blood, your child may receive treatment with additional sugar. Below a certain level of sugar in blood, this is a normal hospital procedure when caring for a sick child. This research study adds that some children with higher levels of sugar in the blood will also be treated with extra sugar.

After your child has been stabilised, we wish to ask some questions to you about your child. We also ask for your permission to read and use information about your child in the clinical notes. We will no intervene in the care of your child except today but would like to follow you during your time at the hospital.

All information we collect about your child will be kept anonymous and will be entered in a database without revealing the name or personal details of your child.

**What are the risks?**

Treatment with sugar intravenously has not shown to constitute any risk as long as the volume given is within a normal range. The venepuncture may cause some discomfort at the site of puncture but is a standard medical procedure for a severely sick child.

**What are the benefits?**

The study will increase the knowledge of when children with severe infections need to be treated with additional sugar.

**What happens if I refuse or want to withdraw later?**

Participating in this study is voluntary and you can refuse now or withdraw your child at any time from the study without giving a reason and with no adverse effect on the care that your child will receive.

**Information**

If you need more information on this research you can contact the study staff and the local study coordinator at site Dr Tim Baker: 1) NUMBER NOT YET AVAILABLE NUMBER

If you want to contact an independent authority about this study you may contact: CONTACT DETAILS NOT YET AVAILABLE

**Consent**

I have read/been read the information for this study and my questions have been properly answered by a member of the research team.

I voluntary agree for my child to participate in the SugaRFACT research study.

Name of child ………………………………………........Signature…..………Date…............... Name of caretaker …………………..………………………..Signature…………...Date…………....

I have explained the study information to the caretaker of this child and have answered the questions

they raised.

Name …………..………………………..............Signature…..………Date…..............

**Witness for patients/mothers who cannot read or write:**

I have witnessed that the caretaker of this child has been read the information for the study and has had his/her questions answered and has agreed to participate in the study.

Name ………………………………………........Signature…..………Date…...............
